# Informal workplaces and their comparative effects on the health of street vendors and home-based garment workers in Yangon, Myanmar: a qualitative study

**DOI:** 10.1186/s12889-020-08624-6

**Published:** 2020-04-19

**Authors:** Thant Ko Ko, Julia Dickson-Gomez, Gisèle Yasmeen, Wai Wai Han, Katherine Quinn, Kirsten Beyer, Laura Glasman

**Affiliations:** 1 Institute for Health and Equity, Medical College of Wisconsin, Milwaukee, USA; 2 Institute of Asian Research, School of Public Policy and Global Affairs, University of British Columbia, Vancouver, British Columbia Canada; 3 Department of Medical Research, Ministry of Health and Sports, No 5, Ziwaka Road, Dagon Township, Yangon, 11191 Myanmar; 4 Center for AIDS Intervention Research (CAIR), Medical College of Wisconsin, Milwaukee, WI USA

**Keywords:** Informal employment, Occupational health and safety, Urban safety, Social determinant of health, Employment relations, Workforce, Low and middle income country, Myanmar

## Abstract

**Background:**

Globally, two billion workers are employed informally but there is limited research on the relationship between informal work and health. Existing studies have focused on informality as an employment condition, with little emphasis on the diversity of physical and social contexts in which informal work takes place. The study considers the diversity of informal workplaces and explores the ways in which this diversity might influence health and well-being of two informal occupational groups in Yangon, the former capital of Myanmar.

**Methods:**

We conducted 21 field observations and 47 semi-structured interviews with street vendors and home-based garment workers based in Yangon, Myanmar. A constant comparative method was used to identify and compare how the physical characteristics of their informal workplaces affect their health for these two informal subgroups.

**Results:**

Although both street vendors and home-based garment workers work informally, their exposure to occupational health and income risks are specific to the physical features of their informal workplaces. Street vendors, who work in public spaces with minimal coverage, are more likely to experience the direct effects of outdoor pollution, inclement weather and ergonomic risks from lifting, carrying and transporting heavy merchandise while home-based garment workers, many of whom live and work in unsanitary housing and deprived neighborhoods, are more likely to experience pollution in or near their homes, and ergonomic risks from poor posture. Similarly, although both groups face safety challenges, street vendors face urban violence and abuse during their commute and at vending points whereas home-based garment workers felt unsafe in their home-based workplaces due to the presence of crime and violence in their neighborhoods.

**Conclusion:**

While informal employment is universally characterized by lack of social protection, exposure to occupational health and income risks for subpopulations of informal workers is determined by the specific physical and social environments of their workplaces. Efforts to improve the health of informal workers should consider the contexts in which informal work takes place to develop tailored interventions for subpopulations of informal workers.

## Background

Informal employment constitutes a significant proportion of the workforce in many low and middle income countries (LMICs) but very little research has studied its effects on health. Decades of research show that pathways linking work and health are complex and operate across multiple levels. Identified pathways encompass both physical and psychosocial influences of employment on health and underscore how they contribute to health patterns both at the individual and population levels [1–3]. Additionally, research shows that income may affect health by directly influencing ability to fulfil health and basic needs essential for biological survival or indirectly determining the degree of social participation and control over professional and personal lives which may affect stress levels [4]. Studies have increasingly focused on the health effects of flexible employment that offers limited employment benefits and social protection [5, 6], finding that they are associated with adverse health outcomes [5, 7–9]. While these studies depict how the changing dynamics of labour markets affect workers’ health, research is limited in LMICs where employment is largely informal and social protection mechanisms are weak or absent.

Globally, approximately two billion workers are informally employed and not covered by statutory social and employment benefits [10]. Many aspects of their work including working conditions, work hours, wages and occupational health and safety (OHS) are unregulated which may have harmful effects on health. However, research on the relationship between informal employment and health in LMICs is scarce. Recent studies have explored how informal work configurations affect health in LMICs and found that informal workers are more likely to report poorer health status [11, 12] and worse quality of life [13] compared to formal workers. Informal work is also associated with a higher risk of experiencing mental illnesses [12, 14]. Similarly, qualitative studies showed that informal workers tend to have poor working conditions, irregular incomes, difficulty accessing health services and are vulnerable to catastrophic health shocks [15–17].

One limitation in existing studies is that they have yet to recognise that informal workplaces are diverse and include various “non-standard” settings such as roadsides, homes and transportation hubs that often lack OHS measures. Such diversity dictates that although some informal occupations may share similar work arrangements, factors influencing workers’ health are likely to vary, depending on their place of work. For instance, while a family business owner who works at home and a street vendor can both be working informally, their work experiences and exposure to OHS risks are likely to differ. However, research investigating how place of work is linked to health for informal workers is limited, except for one study which highlights how place of work contributes to differential exposure to OHS risks among informal workers in Brazil and Ghana [18]. This study explores ways in which the physical work environment affects health and well-being of street vendors (SVs) and home-based garment workers (HBGWs), two informal occupational groups in Yangon, Myanmar. The study has two aims: (1) to describe the informal workplaces of SVs and HBGWs in Yangon; (2) explore and compare ways in which the physical characteristics of their informal workplaces affect SVs and HBGWs’ occupational health and exposure to work-related stress.

### Study context and population

The study took place in Yangon, Myanmar. In Yangon, labour force participation for persons aged 15 years or older was 59.5% for both genders (78.1% for males and 43.3% for females) in 2015 [19]. The overall unemployment rate was 4%, including informally employed workers. Similar to figures in other LMICs, informal employment constitutes approximately three-quarters of the total non-agricultural employment and is the dominant form of employment in Myanmar [19]. In urban areas, approximately 70% of employment is in the informal sector. Yangon has the largest proportion of internal migrants (48.2%), almost 20% of whom reported migrating for work [19].

Since 2011, Myanmar has witnessed an increase in foreign direct investment and industrial expansions [20]. Some sectors such as the garment sector have grown rapidly. The government has initiated labour reforms and passed new laws that seek to offer social security, labour rights and other employment-related benefits including healthcare. Yet, such efforts have largely been limited to formal workers although informally employed workers are the majority in the workforce. In this study, SVs and HBGWs were chosen for comparison because although both groups operate informally, their workplaces differ in terms of workplace characteristics and organisation, regulation, and access to space.

#### Street vendors

In Yangon, SVs who sell a variety of merchandise including food, clothing and other goods are highly visible. The study focused on four downtown townships in Yangon, namely Latha, Lanmadaw, Pabedan and Kyauktada townships. According to data collected in 2016, approximately 6400 SVs operated in these townships [21]. The Yangon City Development Committee (YCDC), the city’s municipal body, is responsible for regulating street vending activities. Street vending in Yangon is considered a marginal economic activity with negative externalities including obstruction to traffic and pedestrian safety, and food safety. However, governance on street vending is focused on limiting, rather than eradicating it.

In 2016, YCDC relocated SVs from the four downtown townships to a designated night market, a roadside vending zone sandwiched between the downtown Strand Road and a raised road by the Yangon river primarily used by container trucks. Concurrently, the government created another vending area near the centrally located Sule Pagoda. Vending spaces were allocated using a lottery system and vending identification cards were issued. Basic facilities such as running water, waste collection and electricity are provided in both sites although weather protection is minimal. In return, SVs pay user fees and taxes. The study participants included both SVs selling in these designated sites and those who continue to sell in other unauthorized public spaces.

#### Home-based garment workers

Home-based workers who produce textiles, artisans and crafts, and other products in home settings are common in LMICs [22]. Home-based workers typically fall into two categories: (1) self-employed and (2) subcontracted. The former incurs the cost of production (buying raw materials, supplies and other costs) and sell their finished goods to local customers whereas the latter (also called homeworkers) produces goods for firms in both local and global markets using materials supplied by their contractors [22]. This study included both types of HBGWs.

Family-owned businesses (mostly unregistered) comprise almost half of the total manufacturing firms in Yangon while micro-firms, defined as enterprises with 1–9 operators, represent 35% of all the enterprises in Yangon [23]. While sub-national level data for sector-specific distribution of firms are not available, informal firms and microenterprises comprise approximately 34 and 70% of the total textile manufacturing firms respectively in Myanmar [23]. This study focused on four townships – namely Hlaingthayar, Shwepyithar, North Okkalapa and Thingungyun townships – where small-scale, home-based garment and textile enterprises are prevalent, and a diversity of housing and neighbourhood settings are observed.

## Methods

This paper presents results from semi-structured interviews with SVs (*N* = 24) and HBGWs (*N* = 23) and 21 ethnographic observations conducted in Yangon, Myanmar between June 2017 and September 2017. The first author conducted observations which lasted about an hour and wrote detailed field notes after each session. To observe SVs, downtown areas were canvassed to select four vending sites where clusters of SVs sell. They were observed four times each (*N* = 16) on varying days and times from a nearby food stall or a street-side café. No consent was sought as the observations were strictly of public behaviour and were non-interactional in nature. However, verbal consent was obtained from HBGWs and other household members prior to observations (*N* = 5) as they took place in their homes. Field notes documented day-to-day operations, workplace physical arrangements (size, density, materials used), working (and living) conditions, neighbourhood characteristics, patterns of social interactions, and potential health impacts of their workplaces. Fieldwork data were triangulated with interviews to obtain additional information about settings and patterns not captured during the interviews. For instance, field notes describing the physical attributes of the informal workplaces were used to contextualise SVs’ and HBGWs’ own accounts of their workplaces and exposure to OHS risks.

Inclusion criteria for both SVs and HBGWs were as follows: (1) being at least 18 years of age at the time of consent, (2) having work experience of at least 3 months in respective occupations, (3) fluent in Burmese; and (4) willing and able to provide verbal consent. Given that home-based garment production is a female-dominant occupation in Myanmar, we expected our final sample to primarily include female participants. Although street vending is a common occupation for both genders, we oversampled women in our study to explore gendered notions of informal work. We used purposive sampling to recruit participants with a diversity of personal characteristics including age, migrant and marital statuses.

Table 1 describes the characteristics of the participants. About two thirds of SVs and HBGWs who participated in the study were internal migrants. The average years of work experience as SVs and HBGWs were 10.6 years and 6.2 years respectively. Most SVs identified themselves as self-employed workers although a diversity of statuses in employment were found among HBGWs. Of the 24 SVs, five self-identified as mobile vendors; nine as semi-itinerant and ten were stationary vendors. Five identified their primary vending location as YCDC-designated vending zones and the rest vended in unauthorized public spaces. All but two HBGWs – one of whom worked in her employer’s home and the other in her neighbour’s home – worked in their own homes. A majority of SVs lived in rental housing, which also was the most common form of housing among HBGWs. However, more than a quarter of the HBGWs in the study lived in informal housing.

**Table 1:** Participant Characteristics

	SV (***N*** = 24)	HBGW (***N*** = 23)
	**Mean [range]**	
**Age (years)**	34.54 [18–57]	36.26 [20–56]
**Length of employment (years)**	10.61 [1–40]	6.24 [0.5–25]
**Number of Children**	1.51 [0–5]	1.3 [0–4]
	**N(%)**	
**Gender**		
Female	16 (66.67)	22 (95.65)
Male	8 (33.33)	1 (4.35)
**Education**		
Elementary or lower	13 (54.17)	5 (21.74)
Middle school	5 (20.83)	7 (30.43)
High school or higher	6 (25)	11 (47.83)
**Marital Status**		
Single	6 (25)	7 (30.43)
Married	15 (62.5)	13 (56.52)
Divorced or widowed	3 (12.5)	3 (13.05)
**Migrant Status**		
Yangon resident	8 (33.33)	8 (34.78)
Migrant	16 (66.67)	15 (65.22)
**Housing Type**		
Rental	17 (71.9)	14 (60.87)
Informal Housing	0 (0)	6 (26.09)
Family-owned	7 (29.1)	3 (13.04)
**Family Type**		
Nuclear	11 (45.8)	11 (47.83)
Extended	13 (54.2)	12 (52.17)
**Employment Status**		
Self-employed	22 (91.67)	10 (43.48)
Wage-employed	1 (4.17)	2 (8.7)
Contributing family Member	1 (4.17)	3 (13.04)
Subcontracted	0 (0)	8 (34.78)

Participants were recruited either directly by the first author who approached potential participants in their workplaces or through referrals from initial participants. A small incentive (2500 Kyats, approximately $2) was offered for study participation. An additional 1000 Kyats ($0.75) was offered for referring up to three participants who were not their family members or relatives. Verbal consent was sought from both groups. During the consent process, participants were explained about the study. They were also assured that their participation in the study is voluntary and will not affect their employment. HBGWs were also explained that their participation in observations will not affect their freedom to participate in interviews. Data collection took place in vending sites (sidewalks, streets, stairwells) and participants’ homes as chosen by the participants.

Study protocols were approved by the Institutional Review Boards at the Medical College of Wisconsin and the Department of Medical Research, Ministry of Health and Sports, Myanmar. Interviews lasted between 45 and 90 min. An open-ended topic guide covering the same content areas for both groups was used (See additional file 1). They included participants’ backgrounds, work history, the process of starting their work and changes over time, employment and workplace arrangements, physical features of and access to their workplaces, factors influencing OHS and income stability, characteristics and safety of the neighbourhoods where they work/live, and self-reported health and quality of life. Identifiable data such as participants’ names and workplace locations were not asked in the interviews.

Digital recordings of interviews were transcribed verbatim and imported into MAXQDA software for coding and analysis. Analysis focused on identification of themes related to the physical contexts of informal workplaces and how they shape workspace arrangements and related OHS and income risks for SVs and HBGWs. A preliminary codebook was developed by open-coding of the field notes and interviews. It was then supplemented with key constructs from the literature. As the analysis progressed, the coding scheme was refined iteratively and continually revised through the addition of emergent codes and themes until the research team agreed that thematic saturation was reached. We used a constant comparative method in our analysis [24]. We first looked for similarities and differences among the two groups separately to explore variability within each group. For instance, we looked for similarities and differences in ergonomic risks among stationary, semi-itinerant and mobile vendors. We then compared SVs and HBGWs in terms of their workplace environments, and workers’ experiences of OHS and income risks. The first author who speaks native Burmese coded all the data. Coded transcripts were reviewed by the fourth author, who also speaks native Burmese, for consistency and accuracy. Discrepancies in coding and interpretations were resolved through regular meetings and a consensus-based team approach. Example quotes were translated into English for group discussions and presented in the findings.

## Results

### Physical features of the workplace

#### Street vendors

A typical workplace for the SVs observed includes a public or semi-public space (e.g. a storefront) located near downtown streets, markets, parks, enclosed alleys and public transportation stations with high pedestrian traffic. The ratio of vendors to available space is high and vendors cluster to occupy space tightly. Street vendors interviewed had varying working hours and days, but many worked from early morning hours (4-6 am) till evening hours (7-8 pm). Some SVs who sold near offices and banks did not work on weekends. Street vendors sell in open air settings, using transportable equipment including baskets, trays, containers, pushcarts, shoulder poles and tables. Street vendors selling food use public space for cooking and preparing food, cleaning, waste collection, storage and serving customers. While many disposed waste and by-products in city-provided bins, trash, overflowing waste bins and wastewater were observed.

The use of space among SVs changes throughout the day. Lacking legal rights to sell in public spaces, SVs share spaces based on mutual understanding and informal rules. Although many SVs occupy “permanent” locations, others canvass the streets on foot. Street vendors carrying shoulder poles or merchandise on their heads frequently stop near busy intersections and roads and sell next to stationary vendors. During the peak hours, this influx of “temporary” vendors adds more density to the already crowded workplaces.

While spatial arrangements vary, one common feature among SVs is the use of cheap makeshift materials such as umbrellas, poles and plastic sheets to reduce operating costs and avoid confiscation and eviction since they are relatively easy to hide and transport to another location. While economical and efficient in avoiding patrolling YCDC officials, they do not protect SVs from physical hazards such as outdoor pollution and inclement weather, which may also cause damage to merchandise. During one observation session, heavy rains blocked some storm drains, causing water to rise and prompting SVs to move their stalls. Others used bricks as weights to prevent their stalls from collapsing due to strong winds and rain. Some also removed the water staying on the nylon coverings that served as temporary roofs and drenched themselves in the process. Throughout the study period which took place during monsoon season, SVs were often seen staying in wet clothes sitting by their stalls.

#### Home-based garment workers

Participants in the study conducted their work in their or their neighbours’ homes, which double as their workplaces. Their work commonly took place in the living rooms or partitioned spaces. The housing types observed included family-owned houses and shared accommodations such as partitioned rooms and dormitory-style rental rooms that accommodate multiple households within a building. Members of the same family typically occupy different rooms within a house although partitioned rooms, frequently observed in rental housing, accommodate multiple families. Several HBGWs reported sharing housing with their landlords.

The house size and the number of co-residents for the HBGWs varied in the study. The number of residents in a household ranged from two to eight. Workplace size varies from a small extension from the landlord’s house used for both living and working purposes (approximately 40 square feet) to residential houses with separate spaces for living and working (approximately 1000 square feet). Besides size, housing quality and amenities also vary, depending on the household income, tenure status and location. Home-based garment workers in mixed-income neighbourhoods tend to live in dwellings constructed with durable materials such as brick, concrete, wood and tin roofs. They usually have access to paved roads, electricity, piped water, private clinics and social services. Others with fewer resources live in low-income neighbourhoods and informal settlements where the houses were built with low-quality makeshift materials such as bamboo, recycled and salvaged materials, cardboard, and thatch roofs that do not provide adequate privacy and safety, and protection from climate conditions. Some houses observed were stilt houses under which wastewater from cooking and household waste such as plastic bottles and discarded rice were observed. In general, the neighbourhoods where low-income participants live often have unsanitary and poor living conditions.

For both occupational groups, the physical features of their workplaces predispose them to OHS risks. These risk factors include environmental pollution, weather and ergonomic risks. Beyond directly influencing physical health, physical work environments lead to income and personal safety risks.

### Environmental pollution

Urban air pollution accounts for 50 to 70 million incidents of respiratory illness in developing countries [25]. Pollution is a serious threat to human health, particularly in overcrowded urban spaces and settlements where the poor in LMICs live and work. For both SVs and HBGWs, their workplaces lack proper infrastructure to protect workers from acute and chronic exposure to environmental pollution.

For SVs, their work activities in open air settings and high traffic areas where they spend long hours outdoors expose them to occupational hazards. They reported that their ability to relocate is constrained by stiff competition for vending spaces and the fear that relocation might hamper their income and loss of an established customer base. Consequently, they accept pollution as an inevitable part of their work.

In the street where I used to sell, there was a storm drain and the lid covering it was usually open…the bad smell was there all the time. [I was there] for 3 years. During the monsoon season, the drain got full and garbage came up to the ground. I had to deal with that. Last year, there was a lot of flooding. **[25-year-old SV, Male]**

Street vendors also complained that they are regularly exposed to urban noise as they sell near other vendors and high vehicular traffic. Since Yangon’s traffic control and regulations are weak, vehicles compete for right of way, leading to incessant honking. Although noise levels vary depending on location, downtown areas tend to have a high concentration of vehicles which creates chronically noisy work environments that may negatively affect their health and quality of life.

When there are traffic jams, I can hear the noise because I’m selling right next to the street. Noises like people angrily honking at other cars and other vendors arguing and fighting or just calling out loud for customers to buy from them. **[24-year-old SV, Female]**

Similarly, many SVs reported exposure to vehicular fumes and pollutants due to heavy traffic congestion in downtown areas of Yangon. The city has seen more vehicles and traffic congestions since 2011 with the liberalization of the automotive industry.

I experience it [exposure to traffic fumes and noise] every day. Sometimes those car owners parked their vehicles near me and sat inside comfortably with the air conditioner on. For us, we suffer from the heat [exhaust fumes]. That’s the life of a street vendor. **[27-year-old SV, Male]**

Another common source of air pollution is cooking outdoors using charcoal, which releases harmful smoke containing toxic chemicals and heat. The use of charcoal as a cooking source is very common in LMICs [26] and linked to respiratory infections, chronic obstructive pulmonary disease (COPD), lung cancer, tuberculosis, asthma, cardiovascular events and all-cause mortality in both adults and children [27]. In Yangon, some SVs, particularly those who prepare made-to-order dishes using large woks or flat pans, use charcoal stoves for cooking outdoors. One SV selling made-to-order *dosa* (Indian pancakes) using charcoal fuel discussed that her daily food preparations cause frequent eye irritations.

I usually keep the fire running until I run out of ingredients […] So the heat [and smoke] comes into my eyes and sometimes I get irritations and swollen eyes and also headaches. People warned that it’s because I stand close to the heat source. **[24-year-old SV, Female]**

She also added that although she has not faced serious health problems, her mother, who started the business and worked for about 20 years, has been experiencing obstructive lung disease, likely from chronic exposure to pollutants from burning charcoal.

Like SVs, HBGWs face OHS risks at their workplaces but the ways in which they are affected differ. As they primarily work indoors, they are less exposed to traffic-related air pollution compared to SVs. Instead, HBGWs face pollution from living in overcrowded, low-quality housing. They identified improper waste disposal and the presence of garbage near their houses as sources of pollution. Because the municipal waste collection system is unreliable, especially in neighbourhoods in the periphery of the city [28], residents typically dispose garbage either at unregulated dumpsites near their houses or within the neighbourhood. For residents living in stilt houses, some choose to conveniently discard their waste under the floor to the ground. This improperly disposed waste accumulates over time, emitting foul smells and creating a breeding space for disease vectors such as rats, flies and mosquitoes.

When it rains, the smell is terrible. My neighbours are so dirty […] they just dump leftovers, rotten food and the polluted water to the ground. Because the houses are close, you can smell it […] when it rains, all the smell comes out. **[52-year-old HBGW, Female]**

Home-based garment workers who live in crowded residential settings are frequently affected by toxic fumes from welding and machinery noise from other home-based enterprises such as metal workshops. Although they did not report experiencing specific illnesses due to these exposures, it has been shown that toxic fumes and particles from welding can negatively affect respiratory health [29].

The bike shop [next to my house] does welding so I frequently get the burning smell. Not all the time but whenever they use welding machine or do metal cutting for a repair work […] There is noise too, but the main problem is that burning smell. **[33-year-old HBGW, Female]**

Besides environmental pollution from proximate sources mentioned above, HBGWs living near factories are affected by pollution including dust, pungent smell from industrial waste, emissions, and side products. As they rely on natural lighting and few houses have proper windows to block undesirable smells, They are constantly exposed to the pollution in both working and non-working hours.

Sometimes, when the alcohol factory dumped their waste, I got bad smell. In the summer, it’s also pungent smell from the fish sauce factory. When it comes, we simply can’t avoid it. It affects the entire neighbourhood…day and night…With that smell, we can’t eat. **[46-year-old HBGW, Female]**

Finally, HBGWs are exposed to dust and particles from the textile materials they use. Chronic exposure to cotton dusts has been shown to increase risks for asthma and COPD and eye problems [30]. For HBGWs in Yangon, this is exacerbated by the crowded living conditions and poorly ventilated workplaces. One HBGW with two decades of work experience shared how it affects her health:

The dust from the garments obstructs my airway. When I spread the garments, it releases small dusts. I couldn’t breathe well. I recovered from it but now it’s back. I need to see a doctor often and she gives me an injection which lasts 3 months […] When it gets worse and I can’t continue [working]. I just take a rest. And when I feel better, I just resume. **[55-year-old HBGW, Female]**

### Weather effects

Weather affects both SVs and HBGWs whose workplaces typically are not properly outfitted for weather protection. Because SVs spend long hours outdoors, weather visibly affects them. Street vendors discussed that heavy monsoon rains in Yangon negatively affect their daily operations from sourcing merchandise at the wholesale markets, setting up stalls to storing unsold items.

Sometimes when the rain is heavy, I have to stay in wet clothes the whole day. Although it’s raining a lot outside, I get up at 3am [and go the wholesale market] to get my merchandise. When it stops raining, my clothes get dry. Then it rains again and they get soaked again. I get sick from that and also get headache and nasal problems. **[25-year-old SV, Female]**

Since transportability is key to a SV’s survival from law enforcement, some vendors see little purpose in adding extra clothes and protective gear to their daily transport. They instead choose to take shelter under the trees, staircases or entrances of private properties during heavy rains and winds. However, they often stay close to the merchandise to prevent damage and theft. One SV explained that he usually prioritises covering up his perishable fruits over himself as he cannot afford to lose his daily capital. For others, they do not use weather protection because it might make them visible to the authorities. Some discussed that when weather is harsh, their protective gear does not provide sufficient protection.

When there is a downpour. I use an umbrella when it’s heavy. But the wind is strong too. Gusty winds […] I can’t use the umbrella. I end up getting drenched in the rain, but I try to cover the fruits. **[24-year-old SV, Female]**

The effects of weather are particularly challenging for some SVs experiencing difficult life transitions such as pregnant SVs or elderly vendors whose health and functional ability are compromised by years of working in poor conditions. Despite these health conditions, they reported that they must continue to work for their survival. While income levels vary among SVs with some reporting earning relatively decent incomes (approximately $200 per month in high-selling seasons), SVs who earn low or erratic incomes from selling several trays of vegetables per day on a small margin do not earn sufficient savings. Thus, they cannot afford taking days off despite their health conditions because they do not have access to social security or sick leave.

My back hurts and my legs also ache [from working]. With my pregnancy, it’s worse. In recent days, my body aches every day. It doesn’t help that I’ve been selling in the rain. I can’t sleep at night. I get back home around 10pm, have dinner and go to bed around 11pm. But I cannot fall asleep easily… I sometimes doubt if I can continue [working] the next day because my body hurts a lot, but I have no choice. It won’t make it easy for me either [if I don’t work]. I need to worry about making my ends meet. **[34-year-old SV, Female]**

Comparatively, HBGWs are less affected by monsoon rains as they work indoors. Their home-based workplaces, while poorly constructed and maintained, at least provide relatively better shelter and weather protection compared to makeshift coverings SVs use in open air. While some HBGWs discussed that heavy rains sometimes cause leaking roofs and flooding, they downplayed the direct effects of monsoon weather on physical health. However, their home-based workplaces present health challenges in the summer when a combination of poor ventilation, high humidity and indoor temperatures, and heat-absorbing construction materials such as tin roofs that trap heat creates unfavourable working conditions. In comparison, while SVs are also affected by the direct sunlight and heat, some shared that their mobile working lifestyles enable them to employ adaptive strategies such as selling under tree shades. For HBGWs, options are limited, and they work in their poorly ventilated workplaces.

I had to be creative and made holes to the [bamboo] wall to make it better. In the summer, I get really sweaty as the room is filled with the pillow stuffing materials [that retain heat]. The only way to relieve this is to bring some air in. **[20-year-old HBGW, Female]**

Weather also poses income risks for both groups by interrupting daily routines and income which in turn increase their stress. Both groups mentioned that monsoon months, from mid-May to September, are associated with lower sales while the ‘dry’ season offers better economic prospects. Most SVs prefer sunny weather which encourages people to spend time outdoors. Monsoon has an opposite effect as it forces customers to stay indoors or away from makeshift stalls. A shoulder-pole vendor complained that rainy weather perennially exposes him to financial uncertainty and affects his ability to pay for housing rent and household expenditures.

I struggle a lot during the rainy season. It’s been a trend for a while now. The sales are meagre, and I borrow money from others […] Summer is usually better because it’s hot and people spend more time outside and buy from me […] in the monsoon season, I use my savings [from the summer] to pay for basic needs at home. **[44-year-old SV, Male]**

In fact, some SVs mentioned that they utilise risk pooling strategies such as renting and sharing a flat (approximately 1000 squared feet) among 15–20 people who came from the same village to afford high rent and living expenses in Yangon. They also reported staying longer hours in poor working conditions when sales are poor. These struggles not only increase their risks for physical health problems but also elevate stress and reduce time spent with family or engaging in social activities that improve their quality of life.

When the rain is heavy, it’s not great for me. I have to stay longer. Sometimes I cannot finish selling everything…I just have to sell it the next day. Sometimes, if I haven’t made my daily income, I just sit and wait whether customers come or not, and only go back home once they are finally sold out. **[34-year-old SV, Female]**

For SVs selling perishable items, they scale down their daily stock to lessen the financial risk. Even with a calculated approach, poor sales still amount to income risks. Not only does weather affect daily sales but it also hampers their ability to reinvest in their daily capital.

When the sales are poor [because of weather], fruits ripen and get rotten quickly. Because I’m selling perishables [there is always a risk]. After one or two days, they change colour and after 3-4 days, you can see the blemishes. Then I have to lower the price or just discard the unsold ones. **[36-year-old SV, Female]**

The stress of dealing with financial uncertainties is amplified when heavy monsoon rains cause flooding in downtown areas which invades SVs’ stalls and makes it difficult for them to continue daily activities. Some vendors cope with this interruption by relocating to less affected areas while others who lack that option halt their work until flooding subsides.

When it [flooding] happens, I move to a different place and leave my shoulder pole …If it’s not raining heavily, I just try to sell. If flooding happens after I’m already out, there is nothing I can do about it. I can’t pack up and go back either so I just sit somewhere. **[25-year-old SV, Male]**

Home-based garment workers also reported experiencing income-related stressful experiences. They discussed that although their home-based workplaces generally offer protection from direct effects of weather during monsoon season, they are also prone to flooding which interrupts their work and reduces income.

When water comes in, I cannot do my work anymore. I made a concrete floor and added bricks to prevent flooding. Even with that, water still came in the other day. The flooding was quite high that day […] When it happened, I couldn’t do work anymore and had to reschedule my order appointments. **[55-year-old HBGW, Female]**

Another way in which weather can affect productivity and income and trigger stress among HBGWs is through power outages, a common occurrence during monsoon season. Both shortage in energy supply and external disturbances caused by severe weather can cause power outages. Typically, weather-induced outages are unpredictable and may last the whole day. While these issues are not limited to neighbourhoods where HBGWs live, they are more likely to experience it frequently because of poorly maintained electric grids in deprived neighbourhoods. As many HBGWs use electric sewing machines, their work is delayed when weather triggers power outages. This is particularly stressful for subcontracted HBGWs who are working on a bulk order that requires working around the clock to meet deadlines.

I usually set a target on how many pieces I want to finish but with power outages it’s challenging. We have to finish 500 pieces this month and this specific design is complex. So, I need to be working pretty much all the time to get it done. Add to that my small child and other household chores, it becomes very stressful. **[34-year-old HBGW, Female]**

Home-based garment workers cope with this by performing other tasks that do not require electricity while some use manual machines or sew by hand. Even for these workers, it is a challenge to work without electricity during monsoon season as natural lighting is inadequate and their work demands precision.

When it rains, the power is gone. Mine is a manual machine so I don’t need electricity. So, I can still do work if there is enough lighting. If the order is not urgent, I stop working but if it is, I use candle light…[it’s] better than just sitting and not doing anything. If I just stop working, I’ll be behind my work…When I do with candle light, it’s not as productive. I have to concentrate more and do it slower to avoid mistakes. **[25-year-old HBGW, Female]**

This quote illustrates how, in addition to increasing work stress, weather-induced disruptions can potentially affect physical health among HBGWs. Some participants discussed that they compensate for the time lost by working well beyond their regular hours (typically 8–10 h per day). When such episodes become frequent, they negatively affect their health by disrupting their sleep patterns and elevating stress levels.

### Ergonomics

Exposure to musculoskeletal risk factors among SVs is linked to their use of transportable materials and ergonomically poor equipment to avoid confiscation and evictions. They often sit on plastic stools or on the ground without back support for extended hours and lift heavy loads on their shoulders or heads. Stationary SVs complained that they frequently experience pain and strains due to sitting in improper posture.

Because I sit all the time, my neck gets tense and I feel drowsy. Sometimes I just take a nap leaning on the long container you see over there. Or if I feel too drowsy, I just sleep anywhere possible. Sometimes, [I nap] on the light truck parked behind me. **[25-year-old SV, Female]**

Shoulder-poles and headload vendors complained that they experience pain due to carrying heavy loads that often include their daily merchandise, utensils for food preparation and in some cases, buckets of cleaning water. While some become used to the pain, chronic pain takes a toll on those doing these activities for years.

One thing I notice is previously I carried things on my head but now I can’t do it anymore. Even for a small load, my head and back hurt and I get pain in my chest. I feel strange. I don’t feel good about myself because I used to be able to do these things. **[51-year-old SV, Female].**

In addition, repetitive and overuse of a body part increases ergonomic risks for some SVs. The aforementioned *dosa* vendor discussed that besides pain from poor posture, her work that requires pouring the batter mixture into the hot pan with one hand while stirring with the other leads to muscle strain due to repetitive motions.

For HBGWs, exposure to ergonomic risks primarily comes from sitting in poor posture or awkward positions such as leaning forward for extended periods without a break.

I don’t feel it [the pain] while working but I feel it when I get up. My legs feel tense and I get neck pain and headache. I usually sit on the floor to cut garments. Perhaps it might be better to do it on a desk. But I have to do it there because the space is small. **[27-year-old HBGW, Female]**

Several HBGWs also reported that they suffer from eye strains, blurred vision and headaches due to a combination of factors including poor posture, sitting for long hours, sustaining high concentration levels and poor lighting.

My neck usually feels tense and I get headaches. I also had eye problems. I couldn’t open them […] My hands hurt too because I cut garments. Also, I have stay focused all the time because the designs are complicated. I also get headache from that. **[50-year-old HBGW, Female]**

### Neighbourhood safety

The physical characteristics of the neighbourhoods where they work influence their perceptions and experiences of safety for both groups and affect their stress levels. For SVs, the scope of workplace neighbourhoods is broad as their day-to-day life often begins at home and involves commuting to wholesale markets and vending sites in downtown neighbourhoods. Because they work long hours almost every day, they barely spend time in their own neighbourhoods. Thus, their experience of neighbourhood safety primarily comes from their daily commute and vending sites. Several female SVs cited that their daily commute in early mornings and late evening hours on poorly lit streets with few pedestrians poses a safety concern. Many SVs choose to go to wholesale markets early in the morning so that they can select from a greater variety of produce. As a safety measure and a cost-effective strategy, they typically commute with fellow vendors. Despite that, several female SVs reported encountering harassment and stressful experiences.

I was waiting at the bus stop with my older sister early in the morning. And there was a driver. It was still dark at that time and he thought we were something [prostitutes]. He made attempts to harass us but luckily there were people nearby and they defended us […] Now we leave a bit later when we can see light. **[18-year-old SV, Female]**

Although the participant downplayed the incident as an unfortunate event, it is clear that such experiences can affect physical and mental health of female SVs who have to commute and work in unsafe neighbourhoods from early morning to late evening hours.

Other SVs mentioned that the presence of police and well-lit downtown neighbourhoods generally discourage criminal activities. Street vendors also sell in clusters to guard themselves against violence and threats. Yet, they occasionally face harassment and verbal abuse from some pedestrians under the influence of substances especially in late evening hours.

I have had some experience with drunk people…One guy asked me how much one basket of strawberries is, and I said 1000-1500 kyats. He then replied, “is it enough? How much is it if I upend the entire stand?” **[32-year-old SV, Male]**

Home based garment workers’ perceptions of neighbourhood safety are primarily based on their residential neighbourhoods. Many HBGWs felt safe working at home but those living in low-income neighbourhoods located on the periphery of the city where law enforcement and public safety measures are weaker mentioned that the prevalence of crime and violence poses safety concerns. These concerns were particularly common among HBGWs whose houses lack proper doors, walls and privacy and are located close to other houses.

In this street, it’s not safe during the monsoon season. There is theft and most people don’t have jobs… a lot of guys also are alcoholics and they keep monitoring other households [to steal things]. **[25-year-old HBGW, Female]**

The same participant discussed that women are at higher risk and how harassment and unwanted approaches from strangers in her own neighbourhood had been part of her life throughout her teenage years to her early 20s until she became married.

Other HBGWs discussed that although they are not personally affected by crime and violence, living and working near street corners and intersections where male causal workers and motorbike-taxi drivers congregate puts them on constant alert regarding their personal safety. They also discussed that frequent news about theft, robbery, fights, murder and harassment in or around the neighbourhoods in which they live affect their perception of safety and heighten their stress.

Because I live close to a street corner, I am worried [about my own safety] … I encounter all kinds of people. Nothing terrible has happened to me so far but I usually see people who are drunk or yelling at others…I try not to go out to dangerous areas in the evening because I hear all the bad things happening. [At night] I block the door with a bike and then set the lock. When I sleep, I have something [a self-defensive tool] next to me. **[46-year-old HBGW, Female]**

Some expressed a desire to relocate to avoid these issues but are also aware that their chances are slim, given the scarcity of affordable housing options elsewhere and potential interruptions to their work. Lacking alternatives, they devise self-defensive strategies in case they face an unfortunate criminal event.

## Discussion

Research indicates that precarious employment, characterised by variable work schedules, lack of a written contract and limited provision of employment benefits, is linked to poor health [6]. Informal work, a form of precarious work, is also linked to negative health outcomes [31]. One limitation of existing studies is that they have not considered how diverse informal workplaces carry different levels of exposure to certain health risks. This study highlights how the physical contexts of informal workplaces (public spaces and homes) differentially affect health and well-being among SVs and HBGWs in Yangon, Myanmar.

Findings in the study are consistent with previous findings on OHS outcomes among SVs and HBGWs in other settings. Studies in other LMIC cities showed that traffic-related air pollutants led to work related illnesses or injuries including upper and lower respiratory symptoms, headache, eye irritations and musculoskeletal problems for SVs [32–34]. A study in Accra, Ghana, found that traffic-related air pollution was positively associated with impaired foetal growth and low birth weight among pregnant SVs. A qualitative study with cross-border women street traders in Accra, Ghana showed that they faced physical health challenges including musculoskeletal pain, swollen feet, body aches, back pain due to long journeys, poor rood networks and working in hazardous work environments [35]. A study based in Phnom Penh, Cambodia, showed that SVs faced personal safety risks in their commute to wholesale markets early in the morning due to theft and robbery [36]. Similarly, our findings on HBGWs are consistent with previous findings. Unhygienic home environments, long working hours and ergonomically poor workplaces were found to pose health and income risks among female home-based workers in India and Pakistan [37]. Similarly, Tipple (2006) found that home-based workers in four different LMIC cities worked for long durations in high intensity work which increased their risk of experiencing poor work-related health outcomes [38].

To date, very few studies have directly compared informal workers in different settings to identify if variations in risk profiles exist across different occupations. To do so is important for three reasons. First, place of work determines the degree of exposure to certain occupational and income risks which may in turn translate into varying levels of vulnerability to specific health outcomes. Second, informal subgroups have different levels of control and autonomy over their workplaces and are subject to different regulations [18] which demands tailored interventions. Third, weak labour institutions in LMICs mean that designing a comprehensive labour protection scheme for all workers including informal workers can be a challenge in practice. Such schemes will likely exclude small-scale informal workers in ‘atypical’ workplaces which could perpetuate their marginal status and poor working conditions. Thus, it is important to recognise the diversity of informal workers and their workplaces and study their characteristics to generate evidence for formulating future interventions and strategies to engage with stakeholders involved in regulation of ‘atypical’ workplaces.

This study identifies “entry points” to intervene to improve the income security, OHS and general health of SVs and HBGWs. Some of these suggestions may be relevant to informal workers in other contexts, although as seen in our results, context matters. First, provision of better infrastructure could promote safer and healthier work environments for both groups. Basic urban services such as clean water, sanitation and storage facilities, proper drainage and waste disposal systems would reduce certain OHS risks in polluted public spaces where SVs work. Efforts to improve the working conditions of street vending in other parts of the world are often accompanied by mass relocation that disrupts regular work patterns including spatial arrangements and income [39]. Due to the unpredictable nature of their urban livelihoods, SVs tend to prioritise income stability over avoiding health risks in choosing their locations. Thus, interventions to improve their health need to take productivity and incomes into consideration. Such provisions are more likely to be sustainable if participatory approaches that involve SVs as stakeholders in decision making processes are employed. For instance, in Bhubaneshwar, India, a multi-stakeholder partnership involving the public and private sectors and SVs co-developed a strategy to finance the project through funding from the private sector who will receive advertising rights in return and build aesthetically pleasing, organised and protected workplaces [40]. A similar financing plan could be an option in Yangon where private actors have already used SVs as an advertising venue. However, it should be acknowledged that not all interventions, however well-intended, may interest or be perceived by SVs as beneficial. Thus, it is imperative that their voices are identified and incorporated in policy dialogues.

Home-based garment workers, especially those living in deprived neighbourhoods and informal settlements, would benefit from improved residential and neighbourhood services including waste disposal and collection systems, proper monitoring and control of industrial pollution, and electricity. Future work should explore the cost effectiveness of different residential upgrading strategies including improving basic physical infrastructure and services (roads, health services), incremental upgrading of shelter, and sustainable strategies that promote residents’ livelihoods and community capital [41]. These efforts can serve as an alternative to relocation, which often contributes to economic instability.

Second, although SVs and HBGWs differ in terms of visibility, they are similar in that they lack access to a formal mechanism to participate in social dialogue with stakeholders who regulate their workplaces because they are unorganised labour. Collective bargaining can help SVs negotiate with the local governments on issues such as the legal use of public space and law enforcement [39]. Similarly, collective bargaining can help HBGWs gain access to better services and infrastructure, credit and skill development trainings, OHS rights and health services. In Thailand, a national home-based worker organisation, HomeNet Thailand, participated in policy dialogues to ensure home-based workers’ access to universal health coverage [18]. However, given the everyday challenges SVs and HBGWs face including long working hours and financial constraints, they may have little opportunity to organise. Non-government organisations and trade unions could act as intermediaries and use their resources to stimulate grassroots movements. Admittedly, one major caveat in following these international examples is that they are from contexts with more advanced and better functioning labour institutions and legal frameworks to protect workers than Myanmar, where labour reforms have only recently begun. Yet, lessons learned from these examples could serve as a starting point in identifying organising strategies for informal workers in Myanmar.

Third, the state’s recognition of SVs’ and HBGWs’ contributions to the economy and the need to improve their working conditions is imperative for expanding OHS rights and social protection to informal workers. National-level policy and legislative reforms are needed to promote their livelihoods and health. These efforts should be complemented by reform at the local level. Local governments directly influence day-to-day livelihood activities of informal workers [18, 42]. In Yangon, SVs in downtown areas are subject to constant monitoring and regulation. Similarly, the local government’s limited action on improving the unsanitary workplaces of home-based enterprises in deprived neighbourhoods due to their informal status negatively affects HBGWs’ health. There should be greater transparency on how local authorities regulate informal work activities and how public resources are allocated for provision of urban infrastructure and services. Furthermore, existing policies should be reviewed to make them better align with on-the-ground economic realities of informal workers.

### Limitations of the study

The study has several limitations. First, the qualitative study design and the small sample size limit assessing the burden of OHS health outcomes. Second, generalisability may be limited because it focuses on four downtown townships for SVs and four industrial townships for HBGWs. For SVs, the social contexts of work may be different for those who operate outside downtown areas where regulation may be less stringent. Similarly, HBGWs working in other parts of the city or in other cities may have different experiences. However, prior studies in other LMIC cities reported similar findings, suggesting that polluted urban spaces and deprived neighbourhoods with low levels of urban services are not unique to Yangon. Future research should recruit a more diverse study population working in different settings to identify potential within-group variations. In addition, findings from this research do not represent the experiences of other informal workers such as waste pickers and day labourers whose informal workplaces are different from those of SVs and HBGWs. The findings are based on the experiences and perspectives of workers and future work should identify the views of stakeholders who regulate informal workplaces.

## Conclusion

This study highlights the importance of considering the diversity of informal workplaces to improve work-related health outcomes among informal workers in LMICs. It shows that approaches that conceptualise informal employment strictly as an employment condition, may not fully capture the notion of informality and should be revisited to incorporate context. Future studies focusing on other informal subpopulations should consider both macro- and micro-social contexts of informal work and workplaces to examine how contextual factors at multiple levels of social influence shape experiences of work and health of informal workers globally.

## Supplementary information

**Additional file 1.** Interview Guide; Open-ended interview guide used for interviewing street vendors and home-based garment workers.

## Data Availability

The datasets generated and/or analysed during the current study are not publicly available due to the possibility of breaching participants’ privacy and confidentiality. In addition, the participants did not consent to make the transcripts of the interviews publicly available. However, excerpts of the interviews are available from the corresponding author on reasonable request.

## Abbreviations

LMIC: Low and middle income country
OHS: Occupational health and safety
SV: Street vendor
HBGW: Home-based garment worker
YCDC: Yangon City Development Committee
ILO: International Labour Organisation
