# Supplementary material for: Informal workplaces and their comparative effects on the health of street vendors and home-based garment workers in Yangon, Myanmar: a qualitative study
Source: BMC Public Health. 2020 Apr 19;20:524. doi: 10.1186/s12889-020-08624-6 (PMC7168952; doi:10.1186/s12889-020-08624-6)
Supplement: Supplementary file 1 — Additional file 1. Interview Guide; Open-ended interview guide used for interviewing street vendors and home-based garment workers. [file 12889_2020_8624_MOESM1_ESM.pdf]

**Interview Guide for In-depth Interviews with Street Vendors**

Thank you for participating in this study. Today I would like to ask questions to explore your experiences and thoughts on your work and health and how your work affects your quality of life and access to healthcare. If you allow, this will be audio-recorded. If you have any questions, you can ask me any time and you are allowed to skip questions if you wish.

First, I would like to ask you a bit about yourself. I will read the questions and you can give short responses.

Age: ______

Gender:
- ☐ female
- ☐ male

Education level:
- ☐ primary school
- ☐ secondary school
- ☐ high school
- ☐ diploma/other training
- ☐ university
- ☐ no education

Are you from Yangon? (migrant status)
- ☐ Yes
- ☐ No

Marital status:
- ☐ single
- ☐ married
- ☐ divorced/separated
- ☐ widowed

Family type:
- ☐ nuclear
- ☐ extended

Number of household members living together: ______

Number of children (if applicable): ______

**I. Personal experiences, daily activities and employment arrangements**

Now I would like to talk about your personal and work experiences.

1. Describe how you got into this job.

- How long have you been working?
- What did you do before?
- *If there was a previous job,*
    - Why did you switch to this job?

    - What are some advantages/disadvantages of your current work compared to the previous one?
    - Overall, what satisfied are you with your current work? why?
- *If from a different region*,
    - How long have you been in Yangon?
    - Why did you decide to move here?
    - Who were your initial connections (family, friends, etc.)?
    - Who do you live with?

2. Is this job your primary source of income? Describe other means (if any) that you support yourself or your family?

- Is your income sufficient for obtaining basic needs (such as food, water, shelter, etc.)?
- Have you ever had trouble having your ends meet? How do you deal with it?

3. Describe you daily routine as a street vendor.

- What are the things you prepare every day?
- Where do you get your merchandize?
- Where do you do the preparation?
- Who else is involved in these daily activities? Tell me more about what they do.
- Do you sell at other places?
- How do you balance work and household duties?

4. Describe your current work arrangement (such as self-employed or employed by another person, subcontracted, etc.)?

*If self-employed,*

- Has it been the same since you started? If not, tell me how you got to this point.
- Does any other person have a stake in this business (such as a family member, a relative or a friend)?

  *If yes,*

    - Tell me about that arrangement.
    - Have you ever had any disagreement with them? Tell me how you deal with it.

*If employed by another person,*

- Describe the arrangement between you and your employer.
- How much choice did you have in deciding the terms?
- What happens if you cannot sell all your merchandize? How does it affect your income?
- How do you get your pay? Have you ever experienced unpaid work days?
- Have you ever lost a job? For what reason? How did you deal with it?

*If selling on commission*,

- How does the arrangement look like?

5. Describe your work schedule on a typical day.

- How many days do you work per week?
- How does it change seasonally? Tell me more about how it varies.
- What are the factors that affect your schedule?

**II. Workplace arrangements**

Now let's move to your business and your current workplace.

6. When you started, what were the things you had to plan?

- Did anyone support you financially or in other ways? Tell me how they helped you.
- What other resources did you need to start?

7. Tell me how you end up in your current vending location.

- What are the things you looked for when you choose a vending site?
- Have you ever been relocated?
  *If relocated*,
  - What did you have to do?
  - Did you get to choose a place or were you assigned one?
  - What are the terms you have to agree in order to keep this place?
  - What changes have you experienced since relocation?

  *If not relocated,*
  - Has your vending location been the same since you started selling? How has it changed over time?
  - How is selling here? Why is it good or not so good? What makes your current location a good place to sell?
  - How did you find this place? Did you find it on your own or through your contacts?

8. Describe how you transport your merchandize.

- What mode of transportation do you usually use?
- Are there any difficulties? Tell me about those.
- Where do you store your things (such as carts, tables and other tools)?

9. How do you keep your regular place? **[If relocated and assigned a regular place, skip to question 9]**

- Do you have to negotiate with others to make sure that your place is not taken? Who do you talk to? Any compromises?
- Have you ever lost your place to others? Describe such a situation. How do you deal with it?
- What other strategies do you use to keep your place?
- Do you have any alternative plans in case your place is taken? Describe them.

**III. Workplace Physical and Social Environments**

10. Describe your current work setting.

- How many customers do you usually get? How does it vary?
- Tell me about your current vending space.
- How about the noise level? How about heat?
- How about smell and pollution (such as dirt, trash and traffic fumes)?
- How do these things affect your health on a daily basis?
- Are there any other factors that we have not discussed?

11. Do you think your workplace is safe? What are the things that make you feel less safe?

- Have you ever experienced bullying or harassment at work? What happened?
- Have you ever witnessed bullying or harassment among other vendors? Tell me what you saw. Did it make you feel concerned about your own safety?

Now let’s talk about your relationships with people near you.

12. Describe your relationships with other sellers.

- Tell me about a situation where you helped them.
- Tell me about a situation where you received help from them.
- Have you ever been involved in a conflict/fight with them? When was the last time? What led to it? How did you resolve it? How did it affect you and your work?
- Is there any seller who, in your opinion, has influence over everyone near you? What makes them dominant?
- When you started here, did you feel like an ‘outsider’? Why? How has it changed since?

13. Describe your interactions with customers.

- What makes a good customer-seller relationship?
- Do you have regular customers? Why do you think they buy from you?
- Have you ever had a situation where you get annoyed by a customer? What happened? How did you deal with it?

14. Describe your interactions with other users of public space (such as pedestrians, cars, property owners, etc.).

*If selling in front of private properties*,

- Did you ask permission from the owners?
- What was their initial impression?
- Do you have any arrangements/understandings with them? What are they?
- Have you ever got into trouble because of them? Tell me about it.
- Tell me some situations (if any) where they help you.

15. In general, what are some good/bad things about your interactions with them?

**IV. Neighbourhood Context**

16. What characteristics make you think this is a good/bad neighbourhood?

- Do you live in the same neighbourhood where you typically sell?

17. Describe the neighbourhood where you live.

- Describe some resources in the neighbourhood that you think are important/helpful.
- Is there any healthcare facility (such as pharmacy, clinics) there?

18. Describe the characteristics of people who live there.

- How is your relationship with your neighbours and others in the community?
- Is there someone you can go to when you need help? How do they help you?
- Do they also come to you when they need help? How do you help them?
- Have you ever thought of moving somewhere? Why?

**V. Law Enforcement**

Most street vendors, at some point in running their businesses, have one or more encounters with authorities (YCDC); some on a daily basis while others infrequently. So I want to talk about it in the next few questions. Before that, I want to reassure you that I have no interaction with them whatsoever and your answers are safe with me. Feel free to skip any question if you do not wish to answer.

19. Describe your experiences and encounters with authorities (YCDC).

- Have you ever been evicted? What happened next?
- How did it affect your income and business? Describe in what ways such encounters affect you.
- How concerned are you that it might happen again?
- Do you think you can prevent it? What strategies do you use?

**VI. Health quality of life, Health Behaviours and Access to Healthcare**

Now let's talk about your thoughts on your life and health. Most questions are about your perception of your health so you do not need to disclose having a disease or other conditions that you do not wish to talk about. If you are not comfortable discussing, you may skip these questions.

20. In general, how satisfied are you with your life?

- Do you think your life is meaningful? Why or why not?
- What are some satisfying things in your life?
- What are some less satisfying things in your life?
- How satisfied are you with your family life?
- Do you think you have freedom to pursue what you want to do? What might be some barriers to do that?

21. Tell me what you satisfied are you with your health.

- Have you ever felt limited due to a physical health condition in your daily life? How does it affect you?
- How you ever felt pain in the past two weeks? Describe your pain.
- Haw satisfied are you with your ability to do daily activities?
- How concerned are you that your physical health might affect your work and income?
- Have you ever taken a break from work due to your health? What happened? Did you seek care for that? How did that affect your income? Who took care of your work while you were absent?
- Have you ever re-adjusted your workload because of your health? When did that change happen?
- Tell me some health problems that affect your daily life.

22. Describe things that make your feel stressful in your daily life.

- How often do you feel stress?
- In general, how do you cope with stress?
- Is there anyone who supports you when you feel down? How do they support you?

23. What happens when you feel sick?

- What is the first thing you do if you are not feeling well?
- Who do you go to or consult with?
- Are there any clinics or doctors nearby?
- How convenient is it to go to a health facility (such as a clinic)?
- Do you think these services are affordable? Why or why not?
- Where do you get your medicine (both western medicine and traditional remedies)?
- How often do you seek medical care when you get sick?

24. Do you have any difficulties in obtaining medical care? Tell me what they are if any.

(probe: transportation, lack of knowledge, cost, work commitment, conflict with other responsibilities, etc.).

- Tell me how these things affect your ability to seek care.
- Have you ever had problems seeking care due to conflict with your work? Describe. How do you deal with it?
- What would you do if you do not feel better?
- How do you usually cope with these difficulties?

25. What is your experience with using healthcare facilities (it could be yourself or with your family)?

- What are some positive/negative experiences?
- What do you think about the quality of services they provide?
- What might prevent you from using them?
- What might make you use them more often?

Do you have any other questions?

Thank you for your participation. I will end the recording here. If you have any questions or concerns, please let me know. Or you can call me at the given number.

**Interview Guide for In-depth Interviews with Home-based Garment Workers**

Thank you for participating in this study. Today I would like to ask questions to explore your experiences and thoughts on your work and health and how your work affects your quality of life and access to healthcare. If you allow, this will be audio-recorded. If you have any questions, you can ask me any time and you are allowed to skip questions if you wish.

First, I would like to ask you a bit about yourself. I will read the questions and you can give short responses.

Age: ______

Gender:
- ☐ female
- ☐ male

Education level:
- ☐ primary school
- ☐ secondary school
- ☐ high school
- ☐ diploma/other training
- ☐ university
- ☐ no education

Are you from Yangon? (migrant status)
- ☐ Yes
- ☐ No

Marital status:
- ☐ single
- ☐ married
- ☐ divorced/separated
- ☐ widowed

Family type:
- ☐ nuclear
- ☐ extended

Number of household members living together: ______

Number of children (if applicable): ______

**I. Personal experiences, daily activities and employment arrangements**

Now I would like to talk about your personal and work experiences.

1. Describe how you got into this job.

- How long have you been working?
- What did you do before?
- *If there was a previous job,*
    - What motivated you to switch to this job?

    - What are some advantages of your current job compared to the previous one?
    - What are some disadvantages?
- *If from a different region*,
    - How long have you been in Yangon?
    - Why did you decide to move here?
    - Who were your initial connections (family, friends, etc.)?
    - Have you been living here since you arrived? Who do you live with?

2. Is this job your primary source of income? If not, describe other means that you support yourself or your family?

- Is your income sufficient for obtaining basic needs (such as food, water, shelter, etc.)?
- Have you ever had trouble having your ends meet? How do you deal with it?

3. Describe your daily routine.

- Do you spend most of your time at home? Is there any work-related activity you do outside of your home? Tell me about it.
- What are the other things you do at home besides your primary work (Probe: childcare, cooking, washing, cleaning, etc.)?
- Who else is involved in these daily activities? Tell me more about what they do.

4. Describe your current work arrangement (such as self-employed or employed by another person, subcontracted or contributing family member, etc.)?

*If self-employed,*

- Has it been the same since you started? If not, tell me how you got to this point.
- Does any other person have a stake in this business (such as a family member, a relative or a friend)?

  *If yes,*

    - Tell me about that arrangement.
    - Have you ever had any disagreement with them? Tell me how you deal with it.

*If employed by another person,*

- Tell me about the arrangement between you and your employer.
- How much choice did you have in negotiating the terms with your employer?
- How do you get your pay? Have you ever experienced unpaid work days?

*If subcontracted work,*

- What are the terms and conditions involved in this arrangement?
- Who are your primary buyers?
- Do orders come directly from a factory (or a shop) or through an intermediary? If both, are there any differences in arrangement between the two? Tell me more about it.
- How much choice do you have deciding the terms? Is it verbally agreed or written down? What do you do if there is a disagreement?
- How do you get paid? (piece rate, daily wage, etc.)
- Do they give you raw materials or do you buy them on your own?
- How strict are the deadlines? What happens if there is a delay on your end?
- Have you ever had delayed payments from your contractors? How did you deal with it?
- What happens if there is an order cancellation? Do you get compensated? How?

*If contributing family member,*

  - Tell me about the arrangement.
  - Do you get paid? How?
  - What other benefits do you have from this work?
  - Do you have any other job besides this?

5. Describe your work schedule on a typical day.

  - How many days do you work per week?
  - How regular is your work schedule? What decides that?

**II. Workplace arrangements and strategies**

Next, let’s talk about your business and your current workplace.

6. How did you (or your family) start this business? What did you need to get started? **[Skip if employed by another person]**

- Did anyone support you financially or in other ways? Tell me how they helped you.
- What other resources did you need to start?

7. Describe how you set up your workspace in your house.

- What are the things you need to do your work?
- What are some good/bad things about the current configuration?
- How does the size of your current workspace affect your productivity?
- How do you store your materials? What arrangements do you have?
- Have you ever had damage to your goods due to current configuration?
- How often do you need to do maintenance to prevent damage to your house?

8. Describe your housing situation (rental, ownership, informal settlement, etc.).

*If rental,*

- How long have you been renting this place?
- What arrangements do you have with your landlord to do your work here?
- How accommodating is your landlord to your needs? Describe.
- Has your landlord ever asked you to stop/postpone your work? For what reason?
- Have you ever made a late payment to your landlord? How did he/she respond to that?
- Have you ever been evicted? When was it? What happened next?
- How concerned are you that you might be evicted in the future?

*If family owned house*,

- Do you need to negotiate with other members of the household? Tell me about the arrangements.

*If informal settlement*,

- Do you need to ask permission from someone? Tell me more about it.
- How concerned are you that you might be forced to move out?

**III. Workplace Physical and Psychosocial Environments**

9. Describe your current living situation.

- How many people are there in your household?
- How many people are involved in your work activities?
- How about sanitation (toilet, sewage, etc.)?
- Does your house have proper ventilation?
- How about lighting (natural light, artificial light)?
- Are you exposed to smell and pollution (such as dust, dirt and waste)?

10. How do you usually do your work (Probe: sitting on the floor, low tables)?

- How do these things affect your health on a daily basis?

11. Do you think your workplace is safe? What are the things that make you feel less safe?

- Have you ever experienced threats or harassment in your workplace or near your house? Tell me about it if you do.

Now let's talk about your relationships with people near you.

12. Describe your relationship with other people at home.

- How supportive are they of your work?
- Is there anyone in your family who disapproves of your work at home? How do you convince them?

- Has your relationship with your household members changed since you started this job? if it has, how has it changed?
- How would you describe your position in the household?
- How much control you have over things in your household?

**IV. Neighbourhood Context**

13. Describe the neighbourhood where you live.

- How long have you been living there?
- What do you think about the safety of this neighbourhood? (Probe: crime, violence)
- How are the streets and houses there?  Are they in good condition?
- Describe some resources in the neighbourhood that you think are important.
- How about healthcare facilities (such as pharmacy, clinics)?

14. Describe the characteristics of people who live there.

- How is your relationship with your neighbours and others in the community?
- Is there someone you can go to when you need help? How do they help you? How reliable are they?
- Do they also come to you when they need help? How do you help them?
- What are some good things you can say about people in your community? Do they get along with each other?
- What are the bad (not so good) things?
- Have you ever felt insecure or stressful because of people in your neighbourhood? Tell me more about such a situation. How do you cope with it?
- Have you ever thought of moving somewhere? For what reason?

**V. Law Enforcement**

15. Have you ever been relocated to another place against your will? Tell me more about it.

- What was the reason behind this relocation? Did you receive warning about it before?
- Did you get any compensation for relocation?
- How did relocation affect your work and income?
- How concerned are you that it might happen again?
- Do you think you can prevent it? How might you prevent it?

**VI. Health quality of life, Health Behaviours and Access to Healthcare**

Now let’s talk about your thoughts on your life and health. Most questions are about your perception of your health so you do not need to disclose having a disease or other conditions that you do not wish to talk about. If you are not comfortable discussing any or all of these questions, you may skip them.

16. In general, what would you rate your quality of life?

- Do you think your life is meaningful? Why or why not?
- What are some satisfying things in your life?
- What are some less satisfying things in your life?
- How satisfied are you with your family life?
- Do you think you have freedom to pursue what you want to do? What might be some barriers to do that?
- What might make your life more satisfying than now? How?

17. Tell me what you satisfied are you with your health.

- Have you ever felt limited due to a physical health condition in your daily life? How does it affect you?
- How you ever felt pain in the past two weeks? Describe your pain.
- How concerned are you that your physical health might affect your work and income?
- Have you ever taken a break from work due to your health? Who took care of your work while you were absent?
- Have you ever re-adjusted your workload because of your health? When did that change happen?
- What are some health problems that affect your daily life?

18. Describe things that make your feel stress in your daily life.

- How often do you feel stress?
- In general, how do you cope with stress?
- Is there anyone who supports you when you feel down? How do they support you?

19. What happens when you feel sick?

- What is the first thing you do if you are not feeling well?
- Who do you go to?
- Are there any clinics or doctors nearby?
- Where do you get your medicine (both western medicine and traditional remedies)?
- How often do you seek medical care when you get sick?
- What prevents you from seeing a doctor?
- How convenient is it to go to a health facility (such as a clinic)?

20. What is your experience with using healthcare facilities (it could be yourself or with your family)?

- What are some positive/negative experiences?
- How would you rate the quality of services they provide?
- What might prevent you from using them?
- What might make you use them more often?

Thank you for your participation in this study. I will end the recording now. If you have any questions or concerns related to this study, please let me know. You can also call the number provided if you have questions later.
